# *Simonsenia aveniformis* sp. nov. (Bacillariophyceae), molecular phylogeny and systematics of the genus, and a new type of canal raphe system

**DOI:** 10.1038/srep17115

**Published:** 2015-11-24

**Authors:** Andrzej Witkowski, Ana Gomes, David G. Mann, Rosa Trobajo, Chunlian Li, Frederik Barka, Evgeniy Gusev, Przemysław Dąbek, Justyna Grzonka, Krzysztof J. Kurzydłowski, Izabela Zgłobicka, Michael Harrison, Tomasz Boski

**Affiliations:** 1 Palaeoceanology Unit, Faculty of Geosciences, University of Szczecin, Mickiewicza 18, 70-383 Szczecin, Poland; 2 CIMA - Centre for Marine and Environmental Research, University of Algarve, Campus de Gambelas, 8005-139 Faro, Portugal; 3 Royal Botanic Garden Edinburgh, Edinburgh, Scotland, UK; 4 Institute of Agriculture and Food Research and Technology (IRTA), Sant Carles de la Ràpita, Spain; 5 Institute for Biology of Inland Waters, Russian Academy of Sciences, Borok, Russia; 6 Institute of Molecular Biosciences, Goethe University, Frankfurt am Main, Germany; 7 Faculty of Materials Science and Engineering, Warsaw University of Technology, Warsaw, Poland; 8 Department of Earth and Atmospheric Sciences, 214 Bessey Hall, Lincoln, NE 68588-0340

## Abstract

The genus *Simonsenia* is reviewed and *S*. *aveniformis* described as new for science by light and electron microscopy. The new species originated from estuarine environments in southern Iberia (Atlantic coast) and was isolated into culture. In LM, *Simonsenia* resembles *Nitzschia*, with bridges (fibulae) beneath the raphe, which is marginal. It is only electron microscope (EM) examination that reveals the true structure of the raphe system, which consists of a raphe canal raised on a keel (wing), supported by rib like braces (fenestral bars) and tube-like portulae; between the portulae the keel is perforated by open windows (fenestrae). Based on the presence of portulae and a fenestrated keel, *Simonsenia* has been proposed to be intermediate between Bacillariaceae and Surirellaceae. However, an *rbc*L phylogeny revealed that *Simonsenia* belongs firmly in the Bacillariaceae, with which it shares a similar chloroplast arrangement, rather than in the Surirellaceae. Lack of homology between the surirelloid and simonsenioid keels is reflected in subtle differences in the morphology and ontogeny of the portulae and fenestrae. The diversity of *Simonsenia* has probably been underestimated, particularly in the marine environment.

Diatoms (Bacillariophyceae) are unicellular photosynthetic organisms with an intricately structured, silicified cell wall^1^. They belong among the Heterokontophyta (stramenopiles), within which they constitute a clade together with the bolidophytes–Parmales^2^,^3^. Diatom evolution extends back to the early Mesozoic, though fossils are rare before the Cretaceous^4^. Through time they have become one of the most important groups of primary producers in aquatic environments^5^ and, with an estimated 100,000 or more species worldwide^6^, they are the second most species-rich group of autotrophic organisms after the flowering plants. Diatoms provide numerous examples of convergence in their evolution. In the past, features such as the presence of a raphe system (a unique organelle possessed by some diatoms, allowing them to glide rapidly over surfaces) on only one of the two valves of the cell wall, have been interpreted as strong evidence for a close phylogenetic relationship. Thus the ‘monoraphid diatoms’ were classified together as a single order, the Achnanthales by Round *et al.* (1990^1^; see also the phylogenetic scheme of Simonsen 1979^7^). Likewise, the development of the raphe system into a canal-like structure, supported beneath by transverse ties of silica (fibulae), was also regarded as a unique evolutionary event^7^. However, with the advent of formal cladistic approaches and molecular phylogenies, it has become clear that neither the monoraphid diatoms nor those possessing a canal raphe constitute monophyletic groups^8^,^9^,^10^. In molecular phylogenies, the monoraphid diatoms, represented by the Achnanthaceae on the one hand, and the Cocconeidaceae and Achnanthidiaceae on the other, have turned out to be distantly positioned, each being related to a different biraphid family^11^,^12^ (Ashworth *et al.* 2013^12^: Fig. S1); recently a third group of monoraphid diatoms has been identified and these cluster together with the Stauroneidaceae^13^. It is clear, therefore, that the loss of the raphe from one valve of each cell in monoraphid diatoms, and from both valves in some other diatoms, e.g. *Diadesmis* and *Diprora*^11^,^14^,^15^, has occurred repeatedly. This convergence could be environmentally forced in relation to habitats where motility has little selective advantage (subaerial crusts, moss carpets, tree trunks, conditions of fast flow where strong attachment to substrata is essential, etc, e.g.^14^,^15^). Similarly, at the ordinal level the canal-raphe bearing diatoms (among which is *Simonsenia*, the focus of the present study) occupy distant positions within the raphid diatoms^10^, showing that fibulae have evolved at least twice.

*Simonsenia* Lange-Bertalot is a genus of motile, raphe-bearing diatoms in which the raphe system opens into a longitudinal canal on a marginal keel^16^. The raphe runs along one margin of the valve and, within a cell, the raphe systems of the two valves are positioned diagonally (‘nitzschioid’ symmetry: for a recent explanation of nitzschioid and hantzschioid symmetry see^17^), as in most *Nitzschia* species. Indeed, in the light microscope (LM), *Simonsenia* species greatly resemble small-celled species of *Nitzschia* sect. *Lanceolatae* and the generitype, *S. delognei* (Grunow) Lange-Bertalot, was originally placed in *Nitzschia* (by Grunow in Van Heurck^18^, p. 184, suppl. pl. C, Fig. 38). It was only when electron microscopy was used to study *S. delognei* that it became evident that the structure of the raphe system differs from that of *Nitzschia* and that, in some respects, it more closely resembles some Surirellaceae. As in some species of *Stenopterobia*, *Surirella* and *Campylodiscus* (e.g.^19^), the raphe canal is elevated on a wing (ala), which is itself fenestrated (i.e. the two sides of the wing fuse at intervals to create external passageways beneath the raphe canal:^1^,^19^,^20^ (Fig. 37e). The raphe canal communicates with the rest of the cell interior only at its ends and via narrow portulae spaced at intervals along the valve. This unexpected discovery by Lange-Bertalot^16^ led him to separate *Nitzschia delognei* into the new genus *Simonsenia*, which he placed in a new subfamily Simonsenioideae Lange-Bertalot within the Nitzschiaceae.

Lange-Bertalot^16^ considered that the structure of the canal raphe was evidence of a “systematic interrelationship between the Nitzschiaceae and the Surirellaceae” but was careful to specify that he did not regard *Simonsenia* as a transitional stage (missing link) between these two large groups [N.B. Bacillariaceae has since been argued to have priority over Nitzschiaceae as the name for a family containing both *Bacillaria* and *Nitzschia*^21^. Subsequently, Ruck and Kociolek^22^ included *S. delognei* as one of the terminal species in a formal cladistic analysis of diatoms possessing a canal-raphe. Their analysis, which was based on morphological characters, supported one of Lange-Bertalot’s conclusions – that *Simonsenia* should be placed in the Bacillariaceae (a conclusion followed by^23^,^24^,^25^,^26^,^27^) – but did not support the idea of a relationship to the Surirellaceae. However, this conclusion has not been tested using molecular phylogenetic approaches.

Until now *Simonsenia* has comprised two species, namely *S. delognei* (Grunow) Lange-Bertalot and *S. delicatula* Mikhailov & Makarova^28^, which is synonymous with *S. delognei* subsp. *rossii* of Lange-Bertalot & Krammer^29^. They are very similar in dimensions and structure, differing only in whether the transapical ribs do (*S. delicatula*) or do not (*S. delognei*) bifurcate near the distal margin of the valve. They cannot be distinguished from each other in LM and so their distributions and ecology are poorly known^30^, although they have been reported from freshwater to slightly brackish-water habitats. Records of either or both *Simonsenia* species have been made in many areas worldwide, including Europe (including the type locality of *S. delognei* in Belgium: Van Heurck^18^), Asia (including the type locality of *S. delicatula* in the Magadan region of the Russian Far East:^28^), North and South Africa, Australia and N America (see^30^); there are also records from S America (e.g.,^31^). However, it is likely that *Simonsenia* species have been largely overlooked or misidentified^30^: because of their small size, marginal raphe and linear-lanceolate outline means, they have probably often been recorded as species of *Nitzschia* sect. *Lanceolatae*. For example, *N. chasei* Cholnoky and *N. atomus* Hustedt are probably misidentifications of *S. delognei* (see Archibald in^16^, see also^30^).

During studies of diatoms from tidal flats close to the river mouth in the estuary of the Guadiana River on the border between Portugal and Spain, we observed a small nitzschioid diatom that did not correspond to any species in the literature. Examination with SEM showed that this diatom possessed the same alar raphe structure as in *S. delognei* and *S. delicatula* (16,28; see also^32^). Fortunately, we were able to isolate a clone of the unidentified *Simonsenia* and maintain it in culture long enough to make preliminary studies of valve morphogenesis and to extract genomic DNA for molecular phylogenetic analysis. The aims of the present study were therefore (1) to document cell and frustule structure, including valve ontogeny, in the new species of *Simonsenia* and provide a formal description of the species, (2) compare the new species with the two previously described *Simonsenia* species, and (3) make a phylogenetic analysis of *Simonsenia* to determine its relationships to Bacillariaceae and Surirellaceae, using the chloroplast-encoded *rbc*L gene, building on earlier phylogenetic analyses of fibulate diatoms (using various genetic markers) by e.g.^10^,^33^,^34^,^35^,^36^,^37^.

## Results

A new species of *Simonsenia* was found in natural population of diatoms collected along the southern coast of the Iberian Peninsula, in the estuaries of the Guadiana (3.85 km from the river mouth –the holotype habitat) and Arade rivers (6.48 and 9.85 km inland from the river mouth). In the Arade estuary its relative abundances ranged from 0.3 to 0.7 %.

### *Simonsenia aveniformis* Witkowski, Gomes and Gusev, sp. nov

**Figure 1:**
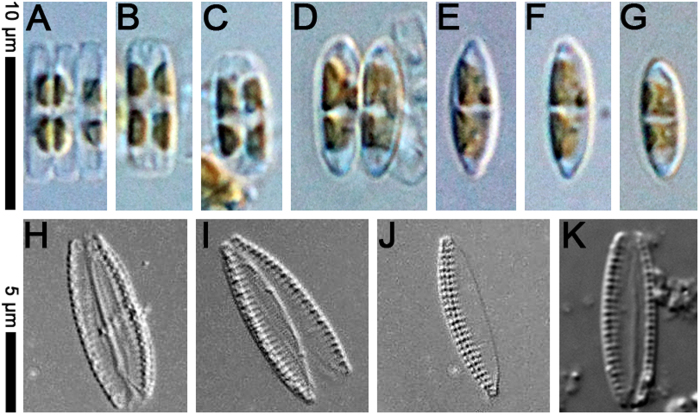
*Simonsenia aveniformis* Witkowski, Gomes & Gusev photographed in the light microscope. (**A–G**) Specimens from the culture to show the type of chloroplasts. (**H–J**) cleaned specimens from the culture. (**K**) cleaned specimen from the natural population sampled in Guadiana River Estuary, site GS1.

**Figure 2:**
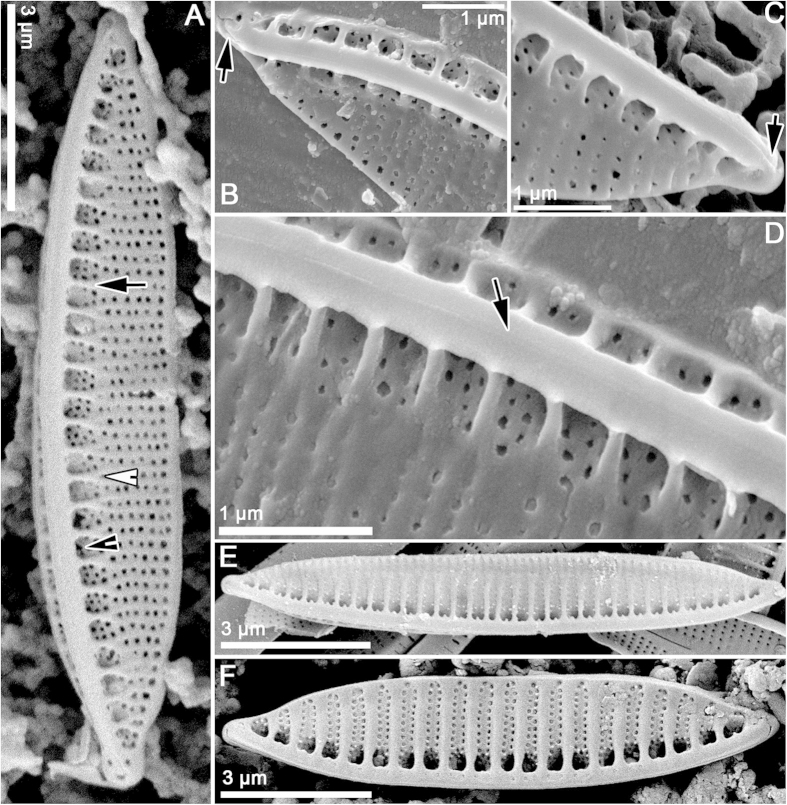
*Simonsenia* spp. in SEM. (**A–F**) External valve views. (**A–D**) External valve views of *S*. *aveniformis*. (**A**) The whole valve with canal raphe elevated above the valve surface. Note the presence of the supporting braces (black arrow) and fenestrae (black arrowhead). The place where the braces join the valve face and the two ribs are formed is marked with a white arrowhead. (**B,C**) close ups of the same specimen: apical parts of valve with apical raphe endings strongly hooked towards the same side (arrow). (**D**) close up of the raphe canal central part demonstrating that raphe slit (arrow) possess no central nodule. (**E**), *S*. *delognei* ssp. *rossii* Lange-Bertalot & Krammer (=*S*. *delicatula* Mikhailov & Makarova) from the holotype sample collected in the Munich Botanical Garden (cf. Lange-Bertalot & Krammer^29^). (**F**), *S*. *delognei*, specimen from Porzeczkowe spring in Central Poland (cf. Witkowski *et al.*^30^).

**Figure 3:**
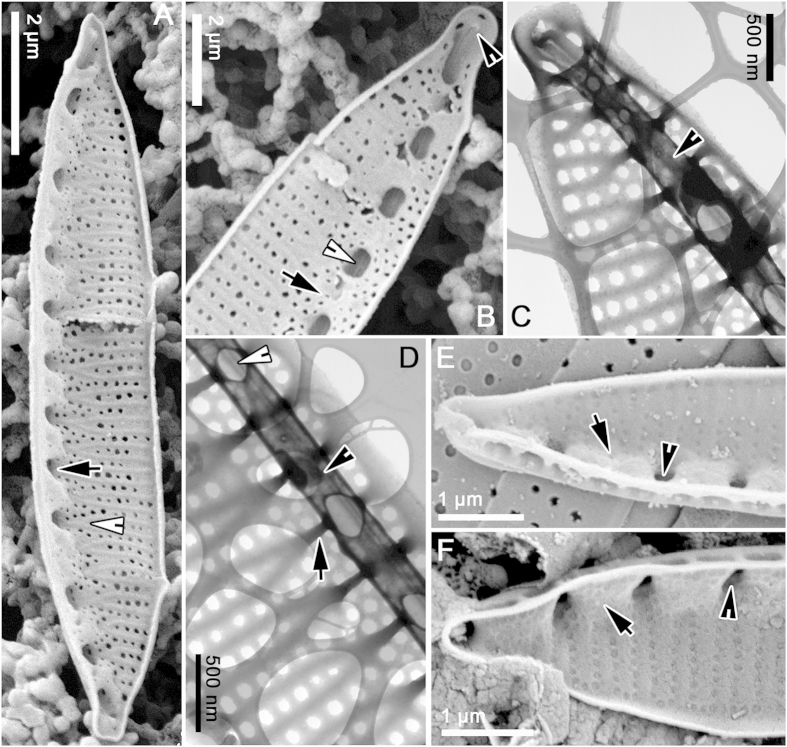
*Simonsenia* spp. in EM. (**A–D**) *Simonsenia aveniformis*. (**A**) *S*. *aveniformis* internal view of the whole specimen, note the internal openings of the portulae = alar canals (arrow) and the place where the two transapical ribs are formed (arrowhead). (**B**) Close up of the apical part of the valve interior, note the presence of the portulae openings (white arrowhead), perforated fibulae (arrow) and of the apical raphe ending terminating in a helictoglossa (black arrowhead). (**C,D**) *S*. *aveniformis* in TEM. (**C**), Valve apical part showing the tube with raphe elevated on braces. Note the elevated raphe bearing tube (arrowhead). (**D**) Close up of the raphe bearing tube with supporting braces composed of solid silica (black arrow), the raphe bearing tube (black arrowhead) and projection of the portulae opening into the raphe bearing tube (white arrowhead). (**E**), *S*. *delognei* ssp. *rossii* close up of the valve interior, note the perforated fibulae (arrow) and the openings of the portulae (arrowhead). (**F**) *S*. *delognei* close up of the valve interior showing perforated fibulae (arrow) and openings of the portulae (arrowhead).

**Figure 4:**
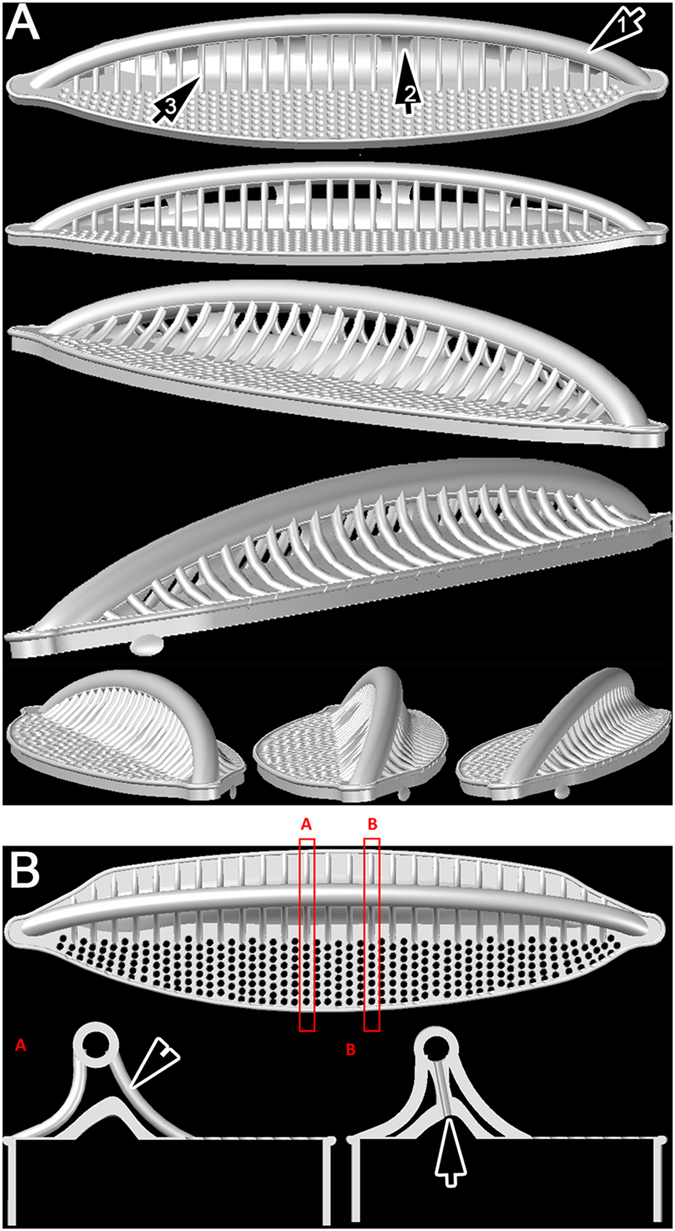
3-D modelling of the simonsenioid raphe system based on FIB cut of numerous specimens from wild population shown at various angles. (**A**) the apical and transapical aspects. Note the presence of the raphe bearing tube (arrow 1), the fenestrae (arrow 2) and the supporting braces (arrow 3). (**B**) Cross sections along transapical axis. Cross section (**A**) shows the position of the raphe bearing canal on braces (arrowhead) above the fibulae, whereas (**B**) shows the connection between the valve interior and the raphe bearing tube through the portulae (arrow).

**Figure 5:**
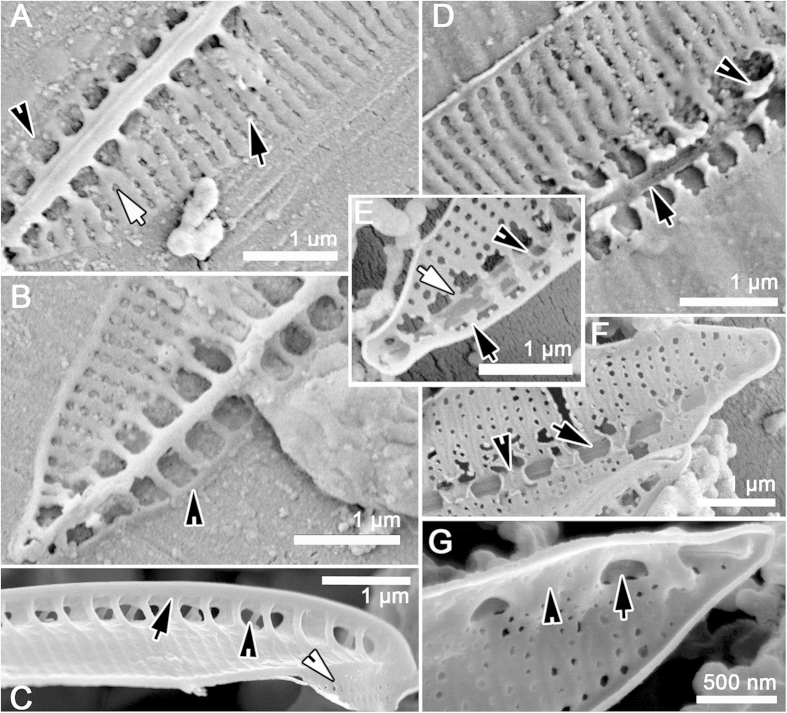
*Simonsenia aveniformis* in a series of SEM images illustrating stages of valve morphogenesis. (**A–C**) External view of the valve formation stages. (**A**) Raphe canal is laid down along with the supporting braces which split into two transapical ribs to form the valve surface structure, note the striae forming areolae still not fully developed (black arrow), the distal and proximal valve margin is still not fused (arrowhead), white arrow marks the place where the brace joins the valve face and is divided into two ribs. (**B**) Close up of the valve apex with raphe canal supported by braces and the transapical striae areolae formed, note the valve margins are formed as continuous strips of silica (arrowhead). (**C**) Specimen from natural population in Guadiana River with well-developed and elevated raphe tube supported by braces (arrow), note the presence of the fenestrae (black arrowhead) and the piece of the girdle band with distinct perforations (white arrowhead). (**D–G**) Valve internal views. (**D**) Raphe canal formed (arrow), the fibulae grow from both sides of the canal forming hammer like structures (arrowhead) are not fully expressed yet. (**E**) Valve interior with more advanced development of fibulae, some hammer like structures connected to span the raphe canal (black arrowhead), the two arrows are marking more advanced development of fibulae but not joined the yet. (**F**) Valve interior with fused fibulae (arrowhead) and cylindrical structures of the initial portulae (arrow). (**G**) Complete valve interior with developed perforated fibulae (arrohead) and portulae (arrow head).

Figures 1, 2, 3, 4, 5, Table 1. DESCRIPTION. Frustules very small, consistently nitzschioid (i.e. with the raphe systems of the daughter cells always formed on opposite sides of the parent following cell division), rectangular and with slightly bevelled corners in girdle view (Figs 1, 2, 3, 4, 5). Each cell with two simple chloroplasts, one located towards each valve end (Fig. 1A–G), both during interphase and during and after cell division (Fig. 1A–D). Valves lanceolate with more or less acute, slightly protracted apices (Fig. 1E–K), 7.5–13.7 μm long, 1.7–2.5 μm broad (*n* = 50). Valve face more or less flat with no transapical undulation (contrast *S. delicatula*); distal mantle almost absent, represented only by a narrow marginal imperforate strip (Figs 2B,D, 3C,D). Raphe almost marginal, without central endings but with bent terminal fissures (Figs 2A–D, 3C,D), appearing in LM as if subtended by rib-like fibulae (Fig. 1H–K), which are revealed by EM to be external braces (fenestral bars) supporting the canal raphe (Fig. 2A–F); there are 24–25 bars in 10 μm. The canal raphe has an apparently nonporous outer wall (Figs 2B–D, 4A–C) and opens to the interior by a series of round portulae (alar canals: Fig. 3A–F), which are separated by rather irregularly porous plates (Fig. 3A–F) functioning as fibulae and as part of the outer wall of the cell. In LM, the portulae are visible only with exceptionally well optimized optics, being much less obvious than the fenestral bars; they also have a significantly lower linear density of ca. 11–12 in 10 μm. In contrast to *S. delognei* and *S. delicatula*, the transapical striae are uniseriate on both the valve face and the proximal mantle and are not resolvable in LM (even with advanced light photomicrography), with ca. 50–60 in 10 μm (Figs 2A–D, 3A–D); transapical ribs (interstriae) not forked near the distal margin (unlike *S. delicatula*). Areolae small, circular, occluded by hymenes (arrowhead in Fig. 5A). Girdle composed of porous bands (arrow in Fig. 5C).

HOLOTYPE: slide no. 19262 housed in the diatom collection of Andrzej Witkowski, at the Palaeoceanology Unit of Faculty of Geosciences at the University of Szczecin (SZCZ), leg. Ana Gomes May 3rd 2011.

ISOTYPES: Coll. Lange-Bertalot (FR) SEM stub B737; Hustedt Collection AWI Bremerhaven, slide no. ZU10/15

TYPE HABITAT: The diatom was collected from sand flats sediments of the Guadiana Estuary (SW Iberia), from a place on the Spanish bank next to the town of Ayamonte (GS1 site: 37°11′41.037″N; 7°24′21.548″W; see Supplementary Dataset 1).

ETYMOLOGY: The specific name is derived from Latin name of oat = *avena*; “*aveniformis*” refers to the similarity of the new species to oat grains as observed under LM.

#### Distribution and autecology

*Simonsenia aveniformis* is only known so far from the estuaries of the Guadiana and Arade Rivers in the Iberian Peninsula (Supplementary Dataset 1). At the sampling sites where *S. aveniformis* was observed, the salinity of the sediment interstitial water ranged between 19.94 (GS1) and 35.71 g/Kg (AS25) while pH varied from 6.77 (AS16) to 7.51 (GS1). The duration of the tidal inundation was between 15.4% (AS25) and 98.9% (GS1). The substratum grain-size varied between fine silt (AS14, AS16 and AS25) and very fine sand (GS1) and the organic carbon and nitrogen percentages ranged from 0.52% (GS1) to 4.25% (AS14) and from 0.06% (GS1) to 0.7% (AS14), respectively. Supplementary Dataset 2 presents in detail the physicochemical parameters measured at each sampling station where *Simonsenia aveniformis* was observed. In the surface waters of the river adjacent to the EI, PT and SF sampling transects, the salinity was similar to that of the sediment interstitial water, varying between 20.58 (SF, low tide) and 32.92 g/Kg (SF, high tide). Regarding pH, river water ranged from 7.8 (SF, low tide) to 8.31 (EI, high tide), the values being slightly higher than those measured in the sediment interstitial water. At sites where *S. aveniformis* was found, dissolved oxygen varied between 83.4% (EI, low tide) and 112.6% (EI, high tide). The temperature of the river water ranged between 17.50°C (EI, high tide) to 18.27°C (SF, high tide).

#### Raphe canal structure

Both cultivated and wild specimens were examined by SEM. A key aspect of *S. aveniformis*, linking it to the two *Simonsenia* described previously, is the structure of the raphe system. Along one side of each valve there is a marginally positioned keel, which is supported externally by solid rib-like braces (fenestral bars; Fig. 2). At its top the keel bears a tubular raphe canal (Figs 2B–D, 3C–D, 5A), which is elevated above the valve face to approximately the same extent over the whole of its length, except at the poles, where the height abruptly decreases and the canal joins rest of the valve. The outer walls of the raphe canal are solid and bear no ornamentation (Figs 2D, 3C,D). The raphe slit is simple and runs along the top of the raphe canal; it is not flanked by ridges. The fenestral bars are not visible internally; instead the course of the raphe canal is marked by a line of small circular portulae and wide fibulae, pierced by areolae like those of the valve face (Fig. 3). The tubular canal raphe is connected with the valve interior only through these portulae and at the apices. Correlated with the absence of central raphe endings, the central portula is no larger than any of the others (Fig. 3A). At the poles the raphe ends internally in a small helictoglossa (Fig. 3A,B), while externally there is a short, bent terminal fissure (Fig. 2B,C).

**Figure 6:**
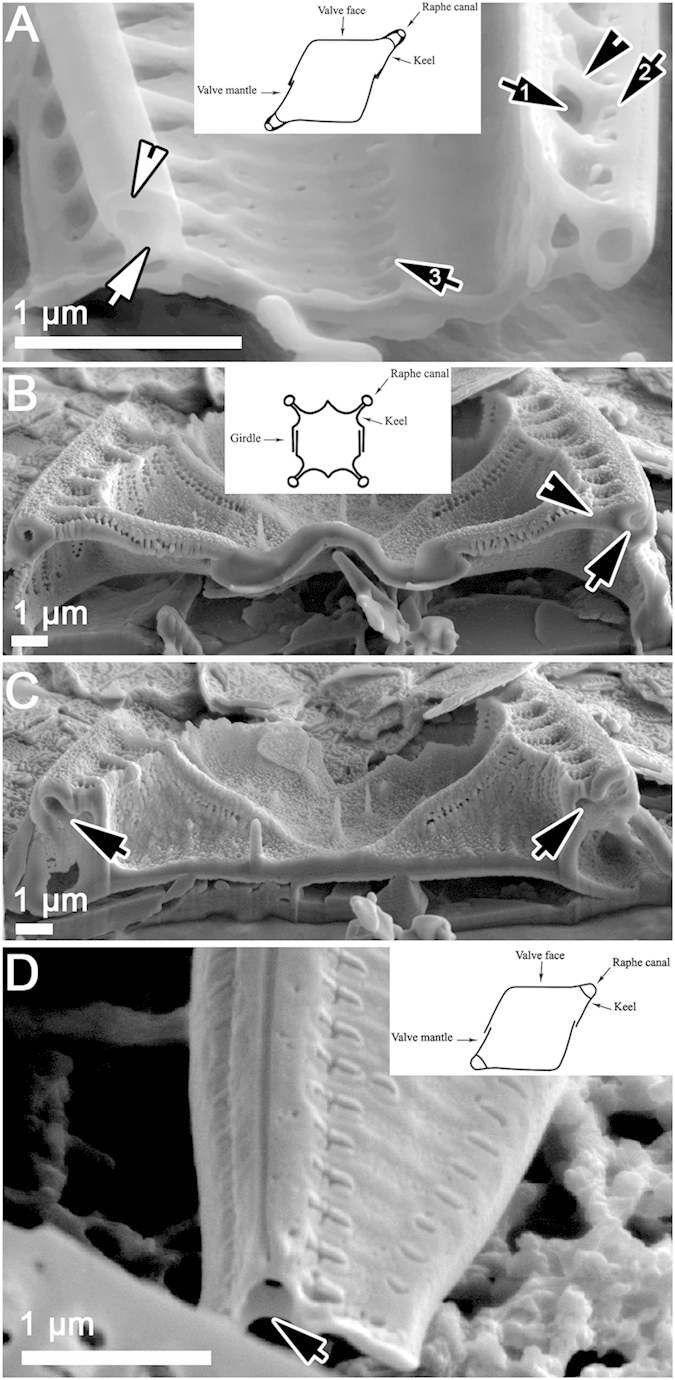
(**A–C**) FIB preparations and diagrammatic presentation of canal raphe bearing systems in diatoms. (**A**) simonsenioid canal raphe: a wild population frustule of *Simonsenia aveniformis* cut in FIB. Note the raphe tube elevated over the valve surface on braces. The left valve shows the opening of a portula (= alar canal, white arrow) connecting the valve with the raphe (white arrowhead) bearing tube. The right valve demonstrates the braces to be solid structures (black arrowhead), and also shows the fenestrae (black arrow 1) and the siliceous membranes sealing the raphe bearing tube in the last stage of the valve morphogenesis (black arrow 2). Black arrow 3 shows the hymenate areolae occlusion. (**B,C**) a surirelloid canal raphe in a wild population *Surirella* sp., cut through the fenestrae. (**B**) Raphe canal (arrow) elevated on braces (arrowhead) showing that raphe tube is incorporated into the whole valve system. (**C**) The raphe system cut to show the portulae (alar canals: arrows). The diagrammatic insert is modified from Ruck & Kociolek^22^. (**D**) nitzschioid canal in *Nitzschia* sp. from wild population. Note the marginal position of the raphe canal. The raphe canal is separated from the valve lumen by a simple fibula (arrow). The diagrammatic insert is modified from Ruck & Kociolek^22^.

The relationship between the fenestral bars and the fibulae and portulae is difficult to visualize, even from SEM images, and we have therefore provided 3-D reconstructions of the valve to aid interpretation (Fig. 4). The external braces (= fenestral bars) that support the raphe canal have almost exactly half the linear density (i.e. they are spaced twice as widely) as the striae, each brace lying opposite and between two of the transapical ribs separating the striae (Fig. 4). Near where each brace arises on the distal side of the raphe, the two transapical ribs opposite the brace converge and fuse (Fig. 4B, but see also 2A, 3A and 5A). Between some of the braces (roughly every second one), the inner wall of the raphe canal extends in towards the main part of the valve and fuses with it, creating the rounded portulae visible from the valve interior in SEM (Fig. 3A,B,E,F) or their openings into the raphe canal tube as seen in Focused Ion Beam (FIB) preparations (Fig. 6A) or in TEM (Fig. 3C,D). Internally, the valve face ribs and striae continue past where the transapical ribs fuse in pairs (i.e. the positions occupied externally by the bases of the fenestral bars) and merge to produce a somewhat irregularly porous fibula beneath the raphe (Figs 3A,B, 5G). Between adjacent portulae (i.e. directly above each fibula), there is a passage (fenestra) outside the cell, connecting valve face to mantle (Figs 5C, 6A). This means that, within the fenestra, the fibula is also the outer wall of the cell.

In SEM preparations of cultured material, we found some valves that had been in the process of formation when the material was fixed. In the earliest stages observed there was almost no sign of the portulae and fibulae: the valve consisted of (1) the raphe, with an incomplete U-shaped, open raphe canal beneath it, and (2) delicate transapical ribs and striae extending to the distal margin (Fig. 5A), though some modelling of the areolae was still ongoing. The only structures linking the raphe to the valve face or to the proximal margin at this stage were the incipient fenestral bars, each of which could be seen to be already linked to two of the transapical ribs on the distal side (i.e. the side where the bars join the valve face); on the proximal side each incipient bar terminated as a simple rib linked to the narrow valve margin (Fig. 6A). Close examination revealed that the incipient bars were differentiated internally into two types: (1) somewhat elevated bars terminating in “hammer-like” structures, and (2) almost flat (slightly concave) strips (Fig. 5D,E). The two types usually alternated, but occasionally hammer-type structures were formed on adjacent ribs. During the following stages of valve ontogeny, the hammer-like structures fused with each other and developed into the fibulae, whereas the flatter strips corresponded to where the portulae would form.

Internally, rows of knob-like projections developed on either side of each fenestral bar (Fig. 5D). Next, the U-shaped canal became modified into a tube, except for a hole – destined to become the portula – left opposite every second bar (arrows, Fig. 5E,F). Portula growth began with development of simple small, cylindrical structures on the inner side of the raphe canal (Fig. 5E), which grew inwards, elevating the raphe canal above the valve face. Through the further growth, branching and proliferation of the knobs, an inner porous wall was created beneath the raphe, forming the fibulae evident in the completed valve (Fig. 5G). This inner wall was continuous with the earlier-formed transapical ribs and striae of the valve face.

#### Molecular phylogeny

**Figure 7:**
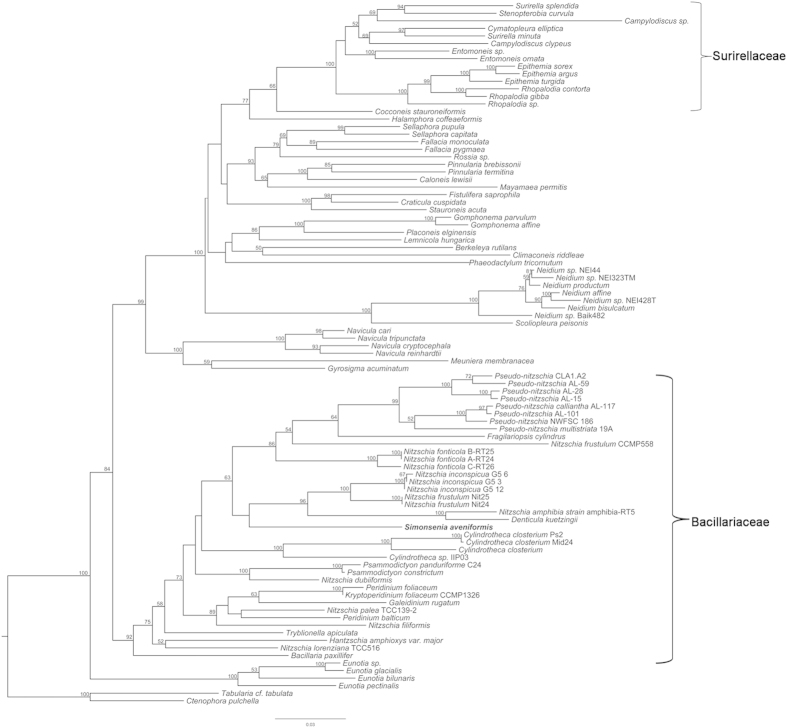
Maximum likelihood phylogeny (with bootstrap values at nodes) inferred from an *rbc*L alignment. *Simonsenia aveniformis*, described in this manuscript, is bolded for clarity. The tree is rooted with the pennate araphid taxa *Ctenophora pulchella* and *Tabularia* cf. *tabulata*. Support values lower than 50% were not included in the tree. The position of the Bacillariaceae and Surirellaceae is marked on the tree.

**Figure 8:**
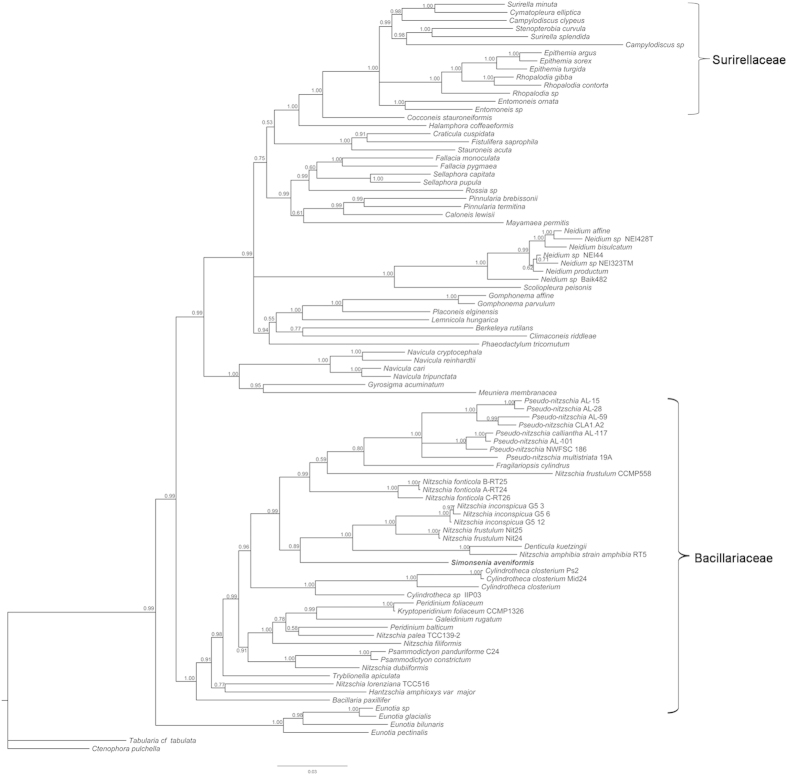
Bayesian Inference phylogeny inferred from an alignment of *rbc*L marker. *Simonsenia aveniformis*, described in this manuscript, is highlighted for clarity. Posterior probabilities are shown at the nodes; nodes with posterior probability lower than 0.50 are collapsed. The tree is rooted with the araphid pennate taxa *Ctenophora pulchella* and *Tabularia* cf. *tabulata*.

The diatom phylogeny was estimated with Maximum likelihood (ML) and Bayesian inference (BI) analysis from 93 taxa (Supplementary Dataset 3) with *rbc*L sequences, using *Tabularia* cf. *tabulata* and *Ctenophora pulchella* as outgroups. In both ML and BI phylogenetic trees (Figs 7, 8), the Bacillariaceae, together with the ‘dinotoms’, fell into one clade, in which *Bacillaria paxillifer* diverged first (bootstrap support [bs] = 92%, posterior probability [pp] = 1); dinotoms are diatom endosymbionts of dinoflagellates^38^,^39^,^40^, in this case the endosymbionts of *Galeidinium rugatum*, *Durinskia baltica* and *Kryptoperidinium foliaceum*. The newly described species *Simonsenia aveniformis* was placed in the Bacillariaceae clade and was sister to a clade containing *Nitzschia inconspicua*, *N. frustulum*, *N. amphibia* and *Denticula kuetzingii* with low support values (bs < 50%, pp = 0.89). This assemblage grouped with a clade composed of *Pseudo-nitzschia* species, *Fragilariopsis cylindrus*, *N. frustulum* and *N. fonticola* (bs = 63%, pp = 0.99). The trees indicated a wide separation between *S. aveniformis* and any members of the Surirellaceae (sensu^1^), which instead were sister to Rhopalodiales (Figs 7, 8). However, in order to test further the evolutionary relationship between *Simonsenia* and the Surirellaceae, which share some characters with *Simonsenia* based on morphology, a constrained analysis was performed, grouping *Simonsenia* in the Surirellaceae. The result showed that the tree with *S. aveniformis* constrained to a clade with the Surirellaceae was significantly worse than the unconstrained tree by a SH test. Hence classification of *S. aveniformis* in or near the Surirellaceae can be rejected.

## Discussion

### Distribution and ecology of *Simonsenia*

It seems that *Simonsenia* is more widespread and species-rich than has previously been thought. Lange-Bertalot^16^ noted that *S. delognei* (which was the only species known until 1983) was almost unrecorded for nearly a century after its original description, but now there are records of it from all continents apart from Antarctica (see Introduction). The discovery of *S. aveniformis* extends the known range of *Simonsenia* into marine habitats, adding another raphid genus to those already known to display evolutionary euryhalinity^41^. Due to the lack of diatom studies along the coastal zone of southern Iberia, *S. aveniformis* remained unknown until now. Here, though it was never abundant, it was present in small numbers in many samples^42^. Probably *Simonsenia* has been under-recorded because of its similarity in LM to small species of *Nitzschia* sect. *Lanceolatae*, which are notoriously difficult to separate and identify (e.g.,^43^). However, examination in SEM reveals a very different structure to that present in *Nitzschia* sect. *Lanceolatae*, where there are no fenestrae in the raphe keel and the fibulae are solid rib- or block like structures.

Table 1 summarizes the morphological differences between *S. aveniformis* and the two previously described species of *Simonsenia* (we synonymize *S. delognei* subsp. *rossii* Lange-Bertalot & Krammer^29^ with *S. delicatula*, following Witkowski^30^, since the diagnostic features of subsp. *rossii* given by Lange-Bertalot & Krammer are present in *S. delicatula*). *Simonsenia aveniformis* is clearly separated from the other two species in LM by the much finer structure of the striae. The major differences between the species, however, are best observed in SEM, and involve contrasting stria and fenestral bar structure. The differences appear to be fewer and less between *S. delognei* and *S. delicatula* than between either of these and *S. aveniformis*.

In terms of autecology, *S. aveniformis* is also distinct from *S. delognei* and *S. delicatula*. *Simonsenia aveniformis* was only observed in sites where the salinity of the sediment interstitial water varied between 19.94 and 35.71 g/Kg (Supplementary Dataset 2), suggesting that its occurrence may be at least influenced by salinity; thus the new species may be classified as a marine to brackish water species. In contrast, *S. delognei* and *S. delicatula* are reported in freshwater to brackish water environments (e.g.,^25^,^28^,^30^,^42^,^44^). It seems that the most favorable habitats for *S. delognei* and *S. delicatula* are riverine supralittoral areas, spring waters^28^,^30^ or the upper reaches of the intertidal zones^42^, whereas *S. aveniformis* inhabits intertidal zones, regardless of the duration of tidal inundation and exposure. Despite its presence in both sandy and silty substrates (Supplementary Dataset 2) *S. aveniformis*, as well as *S. delognei*^30^, reveals a preference for silty sediments, thus it may be classified as a benthic epipelic diatom. According to the data presented by Witkowski *et al.*^30^ and in Supplementary Dataset 2, both *S. aveniformis* and *S. delognei* occurred in environments where the pH of the sediment interstitial water was in the neutral range. *Simonsenia aveniformis*, like *S. delognei*^30^, also reveals high tolerance to pronounced variations in sediment organic carbon (0.52–4.25 %) and nitrogen content (0.06–0.7 %), which may indicate its resilience to organic pollution.

### Systematic position of *Simonsenia*

In this paper we present for the first time a molecular phylogeny of canal raphe bearing diatoms that includes *Simonsenia*. Our results show that the Bacillariaceae and Surirellaceae are not closely related and their fibulate (canal-) raphe systems must have evolved independently. This is consistent with the phylogenetic analysis of Ruck and Theriot^10^, which was based on fewer taxa of canal raphid diatoms than ours but more genes (SSU and *psb*C in addition to *rbc*L). Our results clearly show that *Simonsenia* is unrelated to the Surirellaceae yet it has a similar fenestrated keel to some *Surirella*, *Campylodiscus* and *Stenopterobia* species, which must therefore reflect convergent evolution. It is interesting to examine whether this homoplasy is betrayed by subtle differences in keel structure, or in the way in which the fenestrae and fibulae are formed and this is considered further below (in “Structure and homology of the *Simonsenia* raphe system”).

Classification of *Simonsenia* within the Bacillariaceae is consistent with chloroplast arrangement, since most of the family possess two chloroplasts, one towards each pole (reviewed by Mann^45^), as in *Simonsenia*. Furthermore, in most other Bacillariaceae, like *Simonsenia*, the chloroplasts do not move around in the cell during the cell cycle, retaining their ‘fore-and-aft’ positions during cell division (Fig. 1A–C). It is also consistent with pore structure, since *Simonsenia* species have hymenate pore occlusions (Lange-Bertalot^16^, Lange-Bertalot & Krammer^29^, this paper) like other Bacillariaceae, but unlike the other major groups of canal raphe diatoms, the Rhopalodiales and Surirellales, where the areolae are closed by flaps of silica (e.g.,^46^).

The Bacillariaceae is shown to be monophyletic in our analyses, with good support, but within the Bacillariaceae, the ML and BI phylogenetic trees contain many nodes lacking statistical support, so that the relationships of *Simonsenia* cannot be fully determined. Nevertheless, both analyses indicate that *Simonsenia aveniformis* is located on a long branch and is closely related to a clade containing some species of *Nitzschia* sect. *Lanceolatae* and *Denticula kuetzingii*. Moreover, Bayesian analysis gives good support for a clade containing these taxa with some further members of *Nitzschia* sect. *Lanceolatae* (accessions of *N. fonticola* and *N. frustulum* CCMP558) and also *Pseudo-nitzschia* and *Fragilariopsis.* Hence the genus *Nitzschia* is para- or polyphyletic in our trees and this is consistent with other analyses of the Bacillariaceae that have used *rbc*L (e.g.,^22^,^25^) or other markers (e.g.^33^,^36^) to reconstruct phylogeny.

### Structure and homology of the *Simonsenia* raphe system

Our study shows that, although the molecular phylogeny indicates that the fibulate raphe systems of *Simonsenia* and other Bacillariaceae are essentially homologous, it is obvious that they are also very different, with *Simonsenia* having a much more complicated structure than other Bacillariaceae, with fenestrae in the keel and fenestral bars crossing them (cf. Lange-Bertalot^16^,^29^,^30^,^47^, and this paper). None of the other genera and clades of Bacillariaceae have such a raphe structure. Instead, they have a raphe canal that is integrated into the valve structure, rather than being elevated on a fenestrated wing as in the ‘simonsenioid’ raphe. Furthermore, the fibulae are solid rods, ribs or blocks (e.g.,^1^,^45^), rather than the perforated plates of *Simonsenia*, which function not only as bridges linking the two sides of the valve, but also as part of the external wall of the cell through which it communicates with its environment. The only other Bacillariaceae known to us with similarly porous fibulae to those of *Simonsenia* are some species of *Tryblionella*, e.g. *T. debilis* (Mann^45^, Fig. 730), in which the valve face striae continue without interruption across the interior of the fibula to the proximal mantle. The structure of the keel needs further study in these species, but it is already clear that fenestrations like those of *Simonsenia* are lacking.

Thus, we can distinguish a third type of canal raphe, the “simonsenioid” type, in addition to the “nitzschioid” and “surirelloid” ones recognized hitherto. In our Fig. 6 we provide a summary comparing the three types. This Figure illustrates the principal differences, based on FIB preparations of specimens from natural populations and supplemented with diagrammatic presentations modified from Ruck & Kociolek^22^ in the case of *Nitzschia* and *Surirella*.

The simonsenioid raphe system has converged towards the surirelloid type but the molecular phylogeny shows clearly that it developed from the nitzschioid type. In such cases of homoplasy, detailed inspection sometimes reveals differences that were previously overlooked and we therefore examined the *Simonsenia* raphe for hints of its separate evolutionary origin. At first sight, the canal raphe does seem extremely similar in *Simonsenia* and Surirellaceae, as can be seen by comparing the images of Surirellaceae in Round *et al.*^1^ and Ruck & Kociolek^22^ (particularly those of *Stenopterobia delicatissima* and *S. densestriata*) with those presented here for *Simonsenia aveniformis* (Fig. 6A–C) or by Witkowski *et al.*^30^ for *S. delognei*. However, close examination suggests that there is a difference between the two in that the alar canals and fenestrae of Surirellaceae are formed by undulations (180° out of phase) of the valve face and mantle, which bring the two alternately close together (to fuse and create the fenestrae) and far apart (to create the alar canals). In *Simonsenia*, on the other hand, there are no undulations. This is consistent with the little that is known about ontogeny: in Surirellaceae the fenestrae and portulae are formed by changes in the orientations of the transapical ribs as the ribs extend out from the raphe sternum^48^,^49^, while in *Simonsenia* the raphe canal and portulae are formed after the valve face and mantle structure is essentially complete, if not yet fully thickened, essentially by extension and branching (rather than the orientation) of what become the fenestral ribs in the mature valve.

In contrast to the Surirellaceae, where the question is whether *Simonsenia* has a different raphe ontogeny despite a similar final morphology, in Bacillariaceae the point to examine is whether *Simonsenia* has a similar ontogeny to other genera, even though the mature valves have a very different structure. In *Nitzschia* and *Denticula*, the formation of the valve has been studied and begins with the deposition of the raphe canal, continues with the formation of the transapical ribs and poroids, and is completed by the addition of fibulae, which develop unilaterally, growing across from one side of the valve to fuse with the other side^50^,^51^,^52^. This pretty much resembles our observations in *Simonsenia*, except that in *Simonsenia* the fibulae seem to develop from both sides of the valve simultaneously (via the ‘hammer structures’).

### Taxonomic conclusion

Molecular and morphological results of our study on *Simonsenia aveniformis* and its comparison with established taxa of *Simonsenia* firmly place *Simonsenia* in the family Bacillariaceae. The decision about further subdivisions of the family, i.e. into two sub-families, Bacillarioideae and Simonsenioideae, needs further study and of molecular data in particular. The position of the genus is as follows:

Order: Bacillariales Hendey 1937

Family: Bacillariaceae Ehrenberg 1831

Genus: *Simonsenia* Lange-Bertalot 1979

## Materials and Methods

### Location and site description

*Simonsenia* cells were isolated from samples from the Guadiana and Arade River Estuaries, located in the SW of the Iberia Peninsula. The Guadiana River is the fourth longest in the Iberia Peninsula (with a total length of about 810 km) and the Arade River is the second main river of the Portuguese southern coast (with a length of about 75 km). Both estuaries are under the influence of a Mediterranean climate and experience a semi-diurnal mesotidal regime, with mean tidal amplitude of 2 m. The characteristics of the sites where *Simonsenia aveniformis* was observed and/or isolated (only GS1 site) are provided in Supplementary Datasets S1 and S2.

### Fieldwork

Fieldwork was carried out between 2010 and 2012 in the Guadiana and Arade River Estuaries, yielding the material used here for microscope observations and species culture, and also the environmental measurements (cf. Supplementary Dataset 1 and Gomes *et al.*^53^). Further information concerning the sampling approach and the environmental measurements is available in Gomes *et al.*^53^,^54^.

### Isolation and culturing

To establish clonal cultures, single cells of *Simonsenia* were isolated by micropipette. The captured cells were rinsed several times in sterile medium and finally transferred into wells containing f/2-medium^55^. After 2–3 weeks strains were transferred to plastic Petri dishes and maintained in a cultivation chamber at 22ºC, irradiance of 50 μmol m^–2^ s^–1^ and 12:12 h light:dark cycle for about six weeks. Afterwards the cultures were harvested.

### Microscopy

Light microscopy (LM) and electron microscopy (EM) observations involved the same processing procedure as published in Witkowski *et al.*^56^. LM observations of cleaned material were conducted with a Zeiss Axio Imager 2 (Carl Zeiss, Jena, Germany) with a 100x oil immersion objective (n.a. 1.46). For chloroplast imaging we used the same method as for LM examination of frustules, but in addition, to avoid squashing the cells we placed Tesa tape on the slide (to raise the cover glass and maintain space between the slide and cover glass). As the newly described species has a very small size, we supplemented our own LM observations with a few images kindly taken by ing. Wulff Herwig using his advanced light photomicrography system, for which a detailed description is presented at http://www.microscopy-uk.org.uk/mag/artmar11/Advanced_Light_Photomicrography.pdf.

Ultrastructural analysis was made with scanning and transmission electron microscopy (SEM and TEM, respectively). For SEM examination, a drop of the cleaned sample was filtered onto Whatman Nuclepore polycarbonate membranes (Fisher Scientific, Schwerte, Germany). Filters were air-dried overnight, mounted onto aluminum stubs, and coated with gold-palladium or osmium. SEM observations were made at the University of Frankfurt using a Hitachi S-4500 (Hitachi, Tokyo, Japan) and at the Warsaw University of Technology, Faculty of Materials Science and Engineering, using a Hitachi SEM/STEM S-5500. The observations and deconstruction of *Simonsenia aveniformis*, *Nitzschia* sp. and *Surirella* sp. raphe were performed by means of a Hitachi NB5000 integrated system. The system consists of ultra-high performance Focused Ion Beam (FIB) (40 kV) and high resolution field emission (FE)-SEM (30 kV). This dual beam system enabled high throughput specimen preparation, high resolution imaging and analysis, and precision nanodeconstruction. It enables SEM imaging both during and after FIB deconstruction. The special low-damage deconstruction technique has been applied during diatom processing dedicated for materials sensitive to electron irradiation. The process of deconstruction was performed at accelerating voltage of 10 kV for FIB. The siliceous parts of *Simonsenia aveniformis* raphe were cut at nanoscale and imaged. The diatom specimens have been imaged prior and during particular steps of deconstruction. The deconstruction has been performed on specimens from natural samples. The raphe system was cut primarily along the transapical axis to image the canal supporting system and the connection of canal with the cell lumen via portulae; however, a few cross sections of the canal along the apical axis were made as well.

### Modelling of the raphe system

Model images were generated using the computer aided drafting software AutoCAD. Images of *Simonsenia* were imported into the program in order to derive relative measurements and build a closely approximated basic model. Improvements were made using other images showing various other details and angles. The final model was completed after a number of iterations of the model building process

### DNA extraction, amplification and sequencing

Genomic DNA was isolated from 50 ml of a two-week-old algal cell culture using the Gene Elute™ Plant Genomic DNA Miniprep Kit (Sigma–Aldrich, Hamburg, Germany) according to the manufacturer`s instructions. The chloroplast-encoded gene (*rbc*L) was amplified from genomic DNA using the proof reading polymerase Phusion (Finnzymes, Thermo Scientific, Schwerte, Germany) according to the manual. The primers used for amplification and the protocol of amplification are the same as in Witkowski *et al.*^56^ specified for *rbc*L sequence.

### Phylogenetic analysis

Maximum likelihood (ML) tree of the diatom phylogeny was constructed by means of the program RAxML version7.2.6^57^ with 93 taxa using *Ctenophora pulchella* and *Tabularia* cf. *tabulata* as outgroups. The data, comprising the *rbc*L sequences of *Simonsenia aveniformis* and other *rbc*L sequences downloaded from GenBank (Supplementary Dataset 3) were partitioned by codon position and analysed with the GTR+G+I model. The analysis consisted of multiple runs (20), each with 1000 bootstrap replicates and the tree with the best log likelihood score was chosen as final maximum likelihood estimate. Using the same procedures as for the unconstrained analysis, a constrained ML analysis was performed, forcing *Simonsenia aveniformis* into the Surirellaceae family. The Shimodaira–Hasegawa test^58^ was run using the best constrained tree and the best unconstrained tree from the previous analysis, also using RAxML v 7.2.6 (see Table 2).

For the Bayesian Inference tree, two Bayesian inference analyses each with 4 chains (one cold and three heated), were run with Mr Bayes v.3.2 using the same substitution model and partitioning method as the ML analyses. 10^8^ generations were run per analysis with sampling every 1,000th iteration, generating in total of 10^5^ samples. The final 10^3^ trees were used to get a majority rule consensus tree and obtain posterior probabilities for nodes.

## Additional Information

**How to cite this article**: Witkowski, A. *et al.* *Simonsenia aveniformis* sp. nov. (Bacillariophyceae), molecular phylogeny and systematics of the genus, and a new type of canal raphe system. *Sci. Rep.* **5**, 17115; doi: 10.1038/srep17115 (2015).

## Supplementary Material

Supplementary Dataset 1

Supplementary Dataset 2

Supplementary Dataset 3

## Figures and Tables

**Table 1:** Comparison of *S. aveniformis* with *S. delognei* and *S. delicatula* morphological characteristics.

Characters	*S. aveniformis*	*S. delognei*	*S. delicatula*
Valve length (μm)	7.5–13.7	8–15	12–22
Valve width (μm)	2.0–2.5	1.6–2.0	2–2.5
Transapical ribs (in 10 μm)	50–60	16–22	18–20
Fenestral bars	24-25	16–22	18–20
Fibulae (in 10 μm)	ca. 12	11	8–9^28^
Fenestral bar position	borne from two adjacent transapical ribs	borne on each transapical rib	borne on each transapical rib
Transapical ribs	Simple	simple	branched distally
Striae	Uniseriate	bi- to multiseriate	bi- to multiseriate
Valve face	±flat	±flat	transapically undulate^29^
Structure of the most advalvar bands	two rows of poroids	a single row of poroids	?
Source of information	this paper	see^25^,^30^,^32^	see^28^,^29^

**Table 2:** Results of the Shimodaira–Hasegawa (SH) test for the comparison between the best phylogenetic tree and the constrained phylogenetic tree.

TREE	Likelihood	D(LH)	SD	Significantly Worse
Unconstrained best tree	−52736.129438	−81.615961	23.671871	Yes
Constrained tree	−52817.745400			
