# Supplementary material for: Simonsenia aveniformis sp. nov. (Bacillariophyceae), molecular phylogeny and systematics of the genus, and a new type of canal raphe system
Source: Sci Rep. 2015 Nov 24;5:17115. doi: 10.1038/srep17115 (PMC4656994; doi:10.1038/srep17115)
Supplement: Supplementary Dataset 1 [file srep17115-s1.doc]

***Simonsenia aveniformis* sp. nov. (Bacillariophyceae), molecular phylogeny and systematics of the genus, and a new type of canal raphe system**

Andrzej Witkowski, Ana Gomes, David G. Mann, Rosa Trobajo, Chunlian Li, Frederik Barka, Evgeniy Gusev, Przemysław Dąbek, Justyna Grzonka, Krzysztof J. Kurzydłowski, Izabela Zgłobicka, Michael Harrison, Tomasz Boski

Supplementary Dataset 1. The characteristics of the sites where *Simonsenia aveniformis* was observed and/or isolated (R = River; G = Guadiana River; A = Arade River; ST = Sampling Transect; Elev MSL = Elevation in relation to the mean sea-level; adapted from Gomes, 2013).

| **R** | **ST** | **Sampling sites** | **Coordinates (WGS84)** | | | **Sampling dates** | **Other information** |
| --- | --- | --- | --- | --- | --- | --- | --- |
|  |  |  | **Latitude** | **Longitude** | **Elev MSL** |  |  |
| **G** | **EI** | **GS1** | 37º11'41.037''N | 7º24'21.548''W | -1.325 | 26^th^ May 2010 (first sampling); 3^rd^ May 2011, 14^th^ June 2011, 1^st^ August 2011, 12^th^ September 2011, 11^th^ November 2011, 24^th^ January 2012, 9^th^ March 2012 and 17^th^ September 2012 (sampling for culture) | The site is located in a sand flat of the polyhaline zone of the estuary. Close to the sampling site there is a sewage discharge. |
| **A** | **PT** | **AS6** | 37º9'6.296''N | 8º30'6.979''W | 0.725 | 27^th^ May 2010 | The site is located in a saltmarsh of the polyhaline zone of the estuary. |
|  |  | **AS7** | 37º9'6.299''N | 8º30'6.777''W | 0.628 | 27^th^ May 2010 | The site is located in a saltmarsh of the polyhaline zone of the estuary. |
|  |  | **AS14** | 37º9'6.361''N | 8º30'5.596''W | 0.611 | 27^th^ May 2010 | The site is located in a saltmarsh of the polyhaline zone of the estuary. |
|  |  | **AS16** | 37º9'6.375''N | 8º30'5.192''W | 0.461 | 27^th^ May 2010 | The site is located in a saltmarsh of the polyhaline zone of the estuary. |
|  |  | **AS17** | 37º9'6.390''N | 8º30'4.994''W | -0.148 | 27^th^ May 2010 | The site is located in a mudflat of the polyhaline zone of the estuary. |
|  |  | **AS18** | 37º9'6.396''N | 8º30'4.747''W | -0.331 | 27^th^ May 2010 | The site is located in a mudflat of the polyhaline zone of the estuary. |
|  | **SF** | **AS21** | 37º10'16.409''N | 8º29'15.343''W | 0.264 | 27^th^ May 2010 | The site is located in a mudflat of the polyhaline zone of the estuary. |
|  |  | **AS25** | 37º10'16.187''N | 8º29'14.941''W | 1.057 | 27^th^ May 2010 | The site is located in a saltmarsh of the polyhaline zone of the estuary. |
