# Supplementary material for: Simonsenia aveniformis sp. nov. (Bacillariophyceae), molecular phylogeny and systematics of the genus, and a new type of canal raphe system
Source: Sci Rep. 2015 Nov 24;5:17115. doi: 10.1038/srep17115 (PMC4656994; doi:10.1038/srep17115)
Supplement: Supplementary Dataset 2 [file srep17115-s2.doc]

***Simonsenia aveniformis* sp. nov. (Bacillariophyceae), molecular phylogeny and systematics of the genus, and a new type of canal raphe system**

Andrzej Witkowski, Ana Gomes, David G. Mann, Rosa Trobajo, Chunlian Li, Frederik Barka, Evgeniy Gusev, Przemysław Dąbek, Justyna Grzonka, Krzysztof J. Kurzydłowski, Izabela Zgłobicka, Michael Harrison, Tomasz Boski

Supplementary Dataset 2. Environmental parameters measured in the sampling stations where *Simonsenia aveniformis* has been observed (R = River; G = Guadiana River; A = Arade River; ST = Sampling Transect; TI = duration of the tidal inundation per year; C org = Organic Carbon; DO = Dissolved Oxygen; T = Temperature; HT = High Tide; LT = Low Tide; adapted from Gomes, 2013^42^).

|  |  |  |  | **Sediment** | | | | | | | | **River water** | | | | |
| --- | --- | --- | --- | --- | --- | --- | --- | --- | --- | --- | --- | --- | --- | --- | --- | --- |
| **R** | **ST** | **Sampling sites** | **Salinity (g/kg)** | | **pH** | **TI (%)** | **Grain-size descriptive mean (Folk & Ward Method)** | **Organic content** | | | | **Tidal level** | **Salinity (g/kg)** | **pH** | **DO (%)** | **T (º C)** |
|  |  |  |  |  |  |  |  | **C org**  **(%)** | **CaCO_3_**  **(%)** | **N**  **(%)** | **S**  **(%)** |  |  |  |  |  |
| **G** | **EI** | **GS1** | 19.94 ± 10.02 | | 7.51 ± 0.37 | 98.9 | Very fine sand | 0.52 | 0.20 | 0.06 | 0.05 | **HT** | 30.63 ± 3.86 | 8.31 ± 0.28 | 112.6 ± 13.8 | 17.50 ± 5.64 |
|  |  |  |  |  |  |  |  |  |  |  |  | **LT** | 22.23 ± 6.61 | 8.09 ± 0.23 | 83.4 ± 11.2 | 18.26 ± 5.40 |
| **A** | **PT** | **AS6** | 33.05 ± 4.13 | | 6.89 ± 0.14 | 26.9 | Medium silt | 3.27 | 0.74 | 0.52 | 0.48 | **HT** | 32.99 ± 1.11 | 8.09 ± 0.11 | 100.8 ± 33.2 | 18.15 ± 4.66 |
|  |  | **AS7** | 33.83 ± 3.41 | | 7.15 ± 0.18 | 31.1 | Medium silt | 3.38 | 0.74 | 0.46 | 0.68 |  |  |  |  |  |
|  |  | **AS14** | 29.95 ± 2.68 | | 7.00 ± 0.57 | 31.9 | Fine silt | 4.25 | 0.99 | 0.70 | 0.37 |  |  |  |  |  |
|  |  | **AS16** | 29.11 ± 3.12 | | 6.77 ± 0.39 | 37.6 | Fine silt | 2.89 | 0.62 | 0.50 | 0.21 | **LT** | 25.90 ± 12.83 | 7.89 ± 0.26 | 96.1 ± 25.2 | 17.15 ± 4.74 |
|  |  | **AS17** | 21.64 ± 10.84 | | 7.25 ± 0.06 | 57.3 | Medium silt | 1.28 | 1.25 | 0.20 | 0.12 |  |  |  |  |  |
|  |  | **AS18** | 24.57 ± 12.32 | | 7.34 ± 0.16 | 63.6 | Medium silt | 1.34 | 0.97 | 0.20 | 0.11 |  |  |  |  |  |
|  | **SF** | **AS21** | 26.34 ± 9.57 | | 7.01 ± 0.33 | 45.2 | Medium silt | 2.01 | 0.71 | 0.29 | 0.21 | **HT** | 32.92 ± 2.3 | 7.94 ± 0.14 | 103.8 ± 32.2 | 18.23 ± 5.11 |
|  |  | **AS25** | 35.71 ± 4.14 | | 6.78 ± 0.22 | 15.4 | Fine silt | 4.06 | 1.03 | 0.47 | 0.23 | **LT** | 20.58 ± 5.78 | 7.8 ± 0.24 | 95.1 ± 36.1 | 18.27 ± 4.74 |
