# Supplementary material for: Simonsenia aveniformis sp. nov. (Bacillariophyceae), molecular phylogeny and systematics of the genus, and a new type of canal raphe system
Source: Sci Rep. 2015 Nov 24;5:17115. doi: 10.1038/srep17115 (PMC4656994; doi:10.1038/srep17115)
Supplement: Supplementary Dataset 3 [file srep17115-s3.doc]

***Simonsenia aveniformis* sp. nov. (Bacillariophyceae), molecular phylogeny and systematics of the genus, and a new type of canal raphe system**

Andrzej Witkowski, Ana Gomes, David G. Mann, Rosa Trobajo, Chunlian Li, Frederik Barka, Evgeniy Gusev, Przemysław Dąbek, Justyna Grzonka, Krzysztof J. Kurzydłowski, Izabela Zgłobicka, Michael Harrison, Tomasz Boski

Supplementary Dataset 3. Taxa used to estimate the phylogenetic trees in this paper. *Simonsenia aveniformis* is marked in Bold.

| **Species** | **Strain** | **Genbank Accession** | | |
| --- | --- | --- | --- | --- |
|  |  | **SSU** | ***rbc*L** | ***psb*C** |
| [*Bacillaria paxillifer*](http://www.ncbi.nlm.nih.gov/nuccore/HQ912627.1) (O.F. Müller) T. Marsson | UTEX FD468 | HQ912627 | HQ912491 | HQ912320 |
| *Berkeleya rutilans* (Trentepohl ex Roth) Grunow | ECT3616 | HQ912637 | HQ912501 | HQ912330 |
| *Caloneis lewisii* Patrick | UTEX FD54 | HQ912580 | HQ912444 | HQ912273 |
| *Campylodiscus clypeus* (Ehrenberg) Ehrenberg ex Kützing | L951 | HQ912412 | HQ912398 | HQ912384 |
| *Campylodiscus* sp. | 3613.8 | HQ912413 | HQ912399 | HQ912385 |
| [*Climaconeis riddleae*](http://www.ncbi.nlm.nih.gov/nuccore/HQ912644.1) A.K.S.K. Prasad | ECT3724 | HQ912644 | HQ912508 | HQ912337 |
| *Cocconeis stauroneiformis* (W. Smith) Okuno | s0230 | AB430614 | AB430694 | **---** |
| *Craticula cuspidata* (Kützing) D.G. Mann | UTEX FD35 | HQ912581 | HQ912445 | HQ912274 |
| *Ctenophora pulchella* (Ralfs ex Kützing) D.M. Williams & Round | [UTEX FD150](http://www.ncbi.nlm.nih.gov/nuccore/HQ912611.1) | HQ912611 | HQ912475 | HQ912304 |
| *Cylindrotheca closterium* (Ehrenberg) Reimann & J. Lewin | Ps2 | **---** | JX971009 | **---** |
| *Cylindrotheca closterium* (Ehrenberg) Reimann & J. Lewin | Mid24 | **---** | JX971008 | **---** |
| *Cylindrotheca closterium* (Ehrenberg) Reimann & J. Lewin | CCMP1855 | HQ912645 | HQ912509 | HQ912338 |
| *Cylindrotheca* sp. | IIP03 | **---** | JX971018 | **---** |
| *Cymatopleura elliptica* (Brébisson) | L1333 | HQ912659 | HQ912523 | HQ912352 |
| *Denticula kuetzingii* Grunow | UTEX FD135 | HQ912610 | HQ912474 | HQ912303 |
| *Entomoneis ornata* (Ehrenberg) Ehrenberg | 14A | HQ912411 | HQ912397 | HQ912383 |
| *Entomoneis* sp. | CS782 | HQ912631 | HQ912495 | HQ912324 |
| *Epithemia argus* (Ehrenberg) Kützing | CH211 | HQ912408 | HQ912394 | HQ912380 |
| *Epithemia sorex* Kützing | CH148 | HQ912409 | HQ912395 | HQ912381 |
| *Epithemia turgida* (Ehrenberg) Kützing | CH154 | HQ912410 | HQ912396 | HQ912382 |
| *Eunotia bilunaris* (Ehrenberg) Schaarschmidt | UTEX FD412 | HQ912599 | HQ912463 | HQ912292 |
| *Eunotia glacialis* Meister | UTEX FD46 | HQ912586 | HQ912450 | HQ912279 |
| *Eunotia pectinalis* (Kützing) Rabenhorst | NIES461 | HQ912636 | HQ912500 | HQ912329 |
| *Eunotia* sp. Ehrenberg | ECT3676 | KC309480 | KC309552 | KC309623 |
| *Fallacia monoculata* (Hustedt) D.G. Mann | UTEX FD254 | HQ912596 | HQ912460 | HQ912289 |
| *Fallacia pygmaea* (Kützing) A.J. Stickle & D.G. Mann | UTEX FD294 | HQ912605 | HQ912469 | HQ912298 |
| *Fistulifera saprophila* (Lange-Bertalot & Bonik) Lange-Bertalot | TCC508 | KC736618 | KC736593 | **---** |
| *Fragilariopsis cylindrus* | E8C2 | **---** | EF423499 | EF520304 |
| *Galeidinium rugatum* M.Tamura & T.Horiguchi | **---** | **---** | AB195669 | **---** |
| *Gomphonema affine* Kützing | UTEX FD173 | HQ912608 | HQ912472 | HQ912301 |
| *Gomphonema parvulum*(Kützing) Kützing | UTEX FD241 | HQ912595 | HQ912459 | HQ912288 |
| *Gyrosigma acuminatum* (Kützing) Rabenhorst | UTEX FD317 | HQ912598 | HQ912462 | HQ912291 |
| [*Halamphora coffeaeformis*](http://www.ncbi.nlm.nih.gov/nuccore/KJ463509.1) (C.Agardh) Levkov | 7977-AMPH101 | KJ463449 | KJ463479 | KJ463509 |
| [*Hantzschia amphioxys* var. *major*](http://www.ncbi.nlm.nih.gov/nuccore/HQ912404.1) Grunow | A4 | HQ912404 | HQ912390 | HQ912376 |
| *Kryptoperidinium foliaceum* (F. Stein) Lindemann | CCMP1326 | **---** | GU591328 | **---** |
| *Lemnicola hungarica*(Grunow) F.E. Round & P.W. Basson | UTEX FD456 | HQ912626 | HQ912490 | HQ912319 |
| *Mayamaea permitis* (Hustedt) K. Bruder | TCC540 | KC736630 | KC736600 | **---** |
| *Meuniera membranacea* (Cleve) P.C. Silva in Hasle & Syvertsen | ECT3896 | KC309482 | KC309554 | KC309624 |
| *Navicula cari* Ehrenberg | AT-82.04 | AM501991 | AM710457 | **---** |
| *Navicula cryptocephala* Kützing | UTEX FD109 | HQ912603 | HQ912467 | HQ912296 |
| *Navicula reinhardtii* Grunow | AT-124.15 | AM501976 | AM710442 | **---** |
| *Navicula tripunctata (*O.F. Müller) Bory de Saint-Vincent | AT-202.01 | AM502028 | AM710495 | **---** |
| *Neidium affine* (Ehrenberg) Pfizer | UTEX FD127 | HQ912583 | HQ912447 | HQ912276 |
| *Neidium bisulcatum* (Lagerstedt) Cleve | UTEX FD417 | HQ912591 | HQ912455 | HQ912284 |
| *Neidium productum* (W. Smith) Cleve | UTEX FD116 | HQ912582 | HQ912446 | HQ912275 |
| *Neidium* sp. | NEI44 | **---** | KM078663 | **---** |
| *Neidium* sp. | NEI323TM | **---** | KM078665 | **---** |
| *Neidium* sp. | NEI428T | **---** | KM078666 | **---** |
| *Neidium* sp. | NEIBaik482 | **---** | KM078664 | **---** |
| *Nitzschia amphibia* Grunow | RT5 | **---** | HF675118 | **---** |
| *Nitzschia dubiiformis* Hustedt | s0311 | AB430616 | AB430696 | **---** |
| *Nitzschia filiformis* (W. Smith) Hustedt | UTEX FD267 | HQ912589 | HQ912453 | HQ912282 |
| *Nitzschia fonticola* (Grunow) Grunow | B-RT25 | **---** | HF675067 | **---** |
| *Nitzschia fonticola* (Grunow) Grunow | A-RT24 | **---** | HF675066 | **---** |
| *Nitzschia fonticola* (Grunow) Grunow | C-RT26 | **---** | HF675068 | **---** |
| *Nitzschia frustulum* (Kützing) Grunow | CCMP558 | **---** | EF423498 | EF520303 |
| *Nitzschia frustulum* (Kützing) Grunow | Nit25 | **---** | HF675070 | **---** |
| *Nitzschia frustulum* (Kützing) Grunow | Nit24 | **---** | HF675069 | **---** |
| *Nitzschia inconspicua* Grunow | G5 6 | **---** | HF675092 | **---** |
| *Nitzschia inconspicua* Grunow | G5 3 | **---** | HF675089 | **---** |
| *Nitzschia inconspicua* Grunow | G5 12 | **---** | HF675095 | **---** |
| *Nitzschia lorenziana* Grunow | TCC516 | **---** | KC736608 | **---** |
| *Nitzschia palea* (Kützing) W.Smith | TCC139-2 | **---** | KF959639 | **---** |
| *Peridinium balticum* (Levander) Lemmermann | **---** | **---** | AB195670 | **---** |
| *Peridinium foliaceum* Biecheler | **---** | **---** | UDU31876 | **---** |
| [*Phaeodactylum tricornutum*](http://www.ncbi.nlm.nih.gov/nuccore/HQ912556.1) (Brébisson) W. Smith | CCMP2561 | HQ912556 | HQ912420 | HQ912250 |
| *Pinnularia brebissonii* (Kützing) | UTEX FD274 | HQ912604 | HQ912468 | HQ912297 |
| *Pinnularia termitina* (Ehrenberg) R.M. Patrick | UTEX FD484 | HQ912601 | HQ912465 | HQ912294 |
| [*Placoneis elginensis*](http://www.ncbi.nlm.nih.gov/nuccore/HQ912607.1) (Gregory) E.J. Cox | UTEX FD416 | HQ912607 | HQ912471 | HQ912300 |
| *Psammodictyon constrictum* (W. Gregory) D.G. Mann | s0309 | AB430617 | AB430697 | **---** |
| *Psammodictyon panduriforme* (W. Gregory) D.G. Mann | C24 | **---** | FJ002125 | **---** |
| *Pseudo-nitzschia calliantha* Lundholm, Moestrup & Hasle | AL-117 | **---** | DQ813825 | **---** |
| *Pseudo-nitzschia cuspidata* (Hasle) Hasle | AL-28 | **---** | DQ813820 | **---** |
| *Pseudo-nitzschia delicatissima* (Cleve) Heiden | CLA1.A2 | **---** | EF520341 | **---** |
| *Pseudo-nitzschia dolorosa* Lundhlom & Moestrup | AL-59 | **---** | DQ813822 | **---** |
| *Pseudo-nitzschia hasleana* Lundholm | NWFSC 186 | **---** | JN050304 | **---** |
| *Pseudo-nitzschia mannii* Amato & Montresor | AL-101 | **---** | DQ813824 | **---** |
| *Pseudo-nitzschia multistriata* (Takano) Takano | 19A | **---** | EF423505 | **---** |
| *Pseudo-nitzschia pseudodelicatissima* (Hasle) Hasle | AL-15 | **---** | DQ813817 | **---** |
| *Rhopalodia contorta* Hustedt | L1299 | HQ912406 | HQ912392 | HQ912378 |
| *Rhopalodia gibba* (Ehrenberg) O. Müller | CH155 | HQ912407 | HQ912393 | HQ912379 |
| *Rhopalodia*  sp. | 9vi08.1F.2 | HQ912405 | HQ912391 | HQ912296 |
| *Rossia* sp. | E3333 | EF151968 | EF143281 | **---** |
| *Scoliopleura peisonis* Grunow | UTEX FD13 | HQ912609 | HQ912473 | HQ912302 |
| *Sellaphora capitata* D.G. Mann & S.M. McDonald | BLA11 | **---** | EF143316 | **---** |
| *Sellaphora pupula* (Kützing) Mereschkowsky | BLA14 | **---** | EF143294 | **---** |
| ***Simonsenia aveniformis* Witkowski, Gomes & Gusev** | SZCZ19262 | **---** | **KR048205** | **---** |
| *Stauroneis acuta* W. Smith | UTEX FD51 | HQ912579 | HQ912443 | HQ912272 |
| *Stenopterobia curvula* (W. Smith) Krammer | L541 | HQ912416 | HQ912402 | HQ912388 |
| *Surirella minuta* Brébisson | UTEX FD320 | HQ912658 | HQ912522 | HQ912351 |
| *Surirella splendida* (Ehrenberg) Kützing | 19C | HQ912415 | HQ912401 | HQ912387 |
| *Tabularia* cf. *tabulata* (C. Agardh) Snoeijs | [CCMP846](http://www.ncbi.nlm.nih.gov/nuccore/HQ912615.1) | HQ912615 | HQ912479 | HQ912308 |
| [*Tryblionella apiculata*](http://www.ncbi.nlm.nih.gov/nuccore/HQ912600.1) Gregory | UTEX FD465 | HQ912600 | HQ912464 | HQ912293 |
